# Development of an Indirect Quantitation Method to Assess Ichthyotoxic B-Type Prymnesins from *Prymnesium parvum*

**DOI:** 10.3390/toxins11050251

**Published:** 2019-05-04

**Authors:** Daniel Killerup Svenssen, Sofie Bjørnholt Binzer, Nikola Medić, Per Juel Hansen, Thomas Ostenfeld Larsen, Elisabeth Varga

**Affiliations:** 1 Department of Biotechnology and Biomedicine, Technical University of Denmark, Søltofts Plads 221, 2800 Kgs. Lyngby, Denmark; dankis@bio.dtu.dk; 2 Marine Biological Section, Department of Biology, University of Copenhagen, Strandpromenaden 5, 3000 Helsingør, Denmark; sofie.binzer@bio.ku.dk (S.B.B.); nikola.medic@bio.ku.dk (N.M.); pjhansen@bio.ku.dk (P.J.H.); 3 Department of Food Chemistry and Toxicology, Faculty of Chemistry, University of Vienna, Währinger Str. 40, 1090 Vienna, Austria

**Keywords:** microalgae, haptophyte, phycotoxin, ichthyotoxin, quantitative analysis, HAB, fluorescence detection

## Abstract

Harmful algal blooms of *Prymnesium parvum* have recurrently been associated with the killing of fish. The causative ichthyotoxic agents of this haptophyte are believed to be prymnesins, a group of supersized ladder-frame polyether compounds currently divided into three types. Here, the development of a quantitative method to assess the molar sum of prymnesins in water samples and in algal biomass is reported. The method is based on the derivatization of the primary amine group and subsequent fluorescence detection using external calibrants. The presence of prymnesins in the underivatized sample should be confirmed by liquid chromatography mass spectrometry. The method is currently only partly applicable to water samples due to the low amounts that are present. The growth and cellular toxin content of two B-type producing strains were monitored in batch cultures eventually limited by an elevated pH. The cellular toxin contents varied by a factor of ~2.5 throughout the growth cycle, with the highest amounts found in the exponential growth phase and the lowest in the stationary growth/death phases. The strain K-0081 contained ~5 times more toxin than K-0374. Further investigations showed that the majority of prymnesins were associated with the biomass (89% ± 7%). This study provides the basis for further investigations into the toxicity and production of prymnesins.

## 1. Introduction

**Figure 1:**
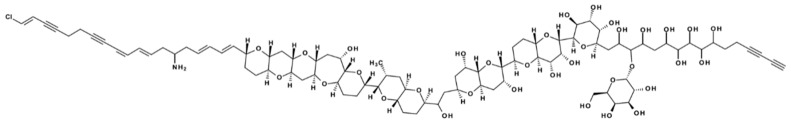
Structure of prymnesin-B2, which was isolated and elucidated by Rasmussen et al. in 2016 [12].

*Prymnesium parvum* is one of the most important harmful algal bloom (HAB) forming species. It has a global distribution and forms fish killing blooms worldwide [1,2]. HAB events caused by *P. parvum* and other HAB forming microalgal species cause devastating economic consequences for the aquaculture in the affected areas as well as for tourism, due to a reduction of angling and other recreational uses of the waters [1,3,4,5]. The mitigation of HAB events is therefore a global matter, and investigations of the causative agents have been going on for many years. Nevertheless, only a few of the causative fish killing agents have been identified (e.g., karlotoxins produced by *Karlodinium veneficum* [6,7] or karmitoxin produced by *Karlodinium armiger* [8]). The causative agents from *P. parvum* have been studied since the 1960s [9], but it was not until the 1990s that the first prymnesins were isolated and their structures elucidated by Igarashi et al. [10,11]. Twenty years later, Rasmussen et al. [12] structure elucidated another type of prymnesins (the B-types, prymnesin-B1 and -B2, shown in Figure 1) and also tentatively identified C-type prymnesins by high-resolution mass-spectrometry (HRMS). The most recent study revealed a total of 51 analogs of prymnesins (A-type: 9, B-type: 12, C-type: 30) [13]. Prymnesins are supersized (1600–2300 Da) ladder-frame polyether compounds, and common for all known prymnesins is the presence of a primary amine [10,11,12]. The current three types (A-, B-, and C-type) are divided based on the number of carbon-atoms in their agylcon backbone structure (A-type: 91, B-type: 85, C-type: 83). Variances within each type are found in the degree of chlorination (one to four), number of sugar moieties attached (zero to three), saturation (up to two extra double bonds) and the number of incorporated oxygen-atoms [13] Table S1 provides an overview of the currently known B-type prymnesins. The different prymnesin types seem to possess slightly different toxicities, with A-type prymnesins being more potent than B-types [12]. Each *P. parvum* strain produces exclusively one type of prymnesin, albeit many analogs within the type and variations between different strains are observed [12,13,14]. 

Quantitative analytical methods for algal toxins that are harmful to humans, like those accumulating in shellfish, are in general well established [15]. However, only few quantitative and semi-quantitative analytical methods have been developed for ichthyotoxic metabolites [16,17,18]. Themost limiting factor in developing analytical methods is the lack of commercially available standards [19] or reliable standards at all. The compounds have to first be isolated from cultures, which is often a huge challenge due to the low natural production, unknown stability and solubility characteristics [18]. Furthermore, some ichthyotoxic metabolites have been described as “sticky”, adhering to different kinds of surfaces (especially plastics), which further challenges purification yields and especially the recovery in analytical methods [16,18,20].

The preferred analytical method for quantitation in complex mixtures is high performance liquid chromatography (HPLC) coupled to mass spectrometry (MS), due to the high selectivity and sensitivity that can be achieved. Quantitation of prymnesins based on MS methods would ideally require purified standards of each analog or, as a bare minimum, they would require a purified standard of one representative analog for each prymnesin type, assuming similar ionization efficiencies within the types. Furthermore, the matrix effects caused by co-eluting matrix components may hamper an accurate quantitation [21]. Using UV/vis-detection would not relieve the need for purified standards of each prymnesin type, while offering less selectivity and sensitivity. Fluorescence detection (FLD) would require an extra step during the sample preparation by adding a fluorescence tag to the prymnesins, for example the primary amine group. Using this approach, a proxy for quantitation (e.g., another primary amine-containing compound like the mycotoxins fumonisin B_1_ or fumonisin B_2_) could be used instead of the purified prymnesin standards. 

The aim of this study was to develop an indirect method for the quantitation of B-type prymnesins. The method was then applied to assess the amount of toxins in the algal biomass as well as the released prymnesins in pH limited batch cultures of two B-type producing *P. parvum* strains (K-0081 and K-0374) during a full growth cycle using a nutrient rich growth medium. 

## 2. Results and Discussion

### 2.1. Extraction of Prymnesins from Biomass

The usual method to extract prymnesins from an algal biomass, applied in our lab, is adapted from Manning et al. [22]. This method uses centrifugation to separate the biomass from the water, followed by a two-step extraction of the biomass: (1) cold acetone removes chlorophyll, among other compounds, and (2) methanol (MeOH) extracts the prymnesins [12,13]. For this study, the centrifugation step was replaced by a gentle filtration using glass microfiber filters to ease the handling of large volumes and to minimize the cell friction (as shown in Section 2.5). Another necessary modification was to skip the removal of chlorophyll with acetone, as the water present on the filter material increased the solubility of the prymnesins during this extraction, resulting in prymnesin losses. A series of pre-experiments was conducted to determine the amount of biomass needed for quantitative purposes. For the *P. parvum* K-0081 strain, 9 million cells were sufficient, while 60 million cells were required for the K-0374 strain, due to differences in the amount of produced prymnesins under the chosen conditions. 

For validation purposes, unused filters were spiked with prymnesin extracts, processed according to the developed protocol described in Section 4.7 and compared to a reference sample (prymnesin extract diluted in MeOH). The apparent recovery was determined to be 58% with a relative standard deviation (RSD) of 7%. Further investigations showed that approximately 75% of the loss was due to the drying and reconstitution step, and no significant differences between the polypropylene tubes and glass vials were observed. Future improvements of the method should focus on enhancing the recovery of prymnesins during the reconstitution step. Still, the apparent recovery was comparable to that achieved for similar compounds, e.g., karmitoxin [18]. 

### 2.2. Quantitation via Fluorescence Tagging of Prymnesins in the Biomass Extract

Due to the lack of standards, the quantitation of prymnesins by UV- or MS was not possible. However, the common denominator of all currently known prymnesins is the presence of a primary amine. This feature was used in quite an unspecific derivatization procedure attaching a fluorescence tag and using two other primary amine containing compounds (fumonisin B_1_ and B_2_) as a proxy for the quantitation. Since the molecular mass varies considerably (1655 Da to 2015 Da), the prymnesin content is expressed as a molar sum. The repeatability of the fluorescence tagging method was assessed by investigating the relative standard deviation of three individually derivatized samples from the same extract, showing acceptable values of 3.5% for the extracts of strain K-0081 and 5.6% for strain K-0374. The theoretic limits of the method were determined using the calibration curve method [23]. A limit of quantitation (LOQ) of 52 µmol/mL was obtained, while the limit of detection (LOD) was 16 µmol/mL (*n* = 10). However, these limits corresponded to peak areas under one, the minimum requirement for integration by the software (ChemStation Rev. B01.01). Thus, a more realistic LOQ based on the calculated concentration of a peak area of a value over one is 80 µmol/mL in the measurement solution. Taking into account the dilution factor of the derivatization step (usually 5) and assuming a prymnesin concentration of ca. 140 attomol/cell in the case of strain K-0081, these are approximately 3.2 million cells. For strain K-0374, which had a lower prymnesin content per cell (ca. 30 attomol/cell), the required number is approximately 15 million cells. The peak area may be increased by increasing the injection volume from 10 to 20 µL or by reducing the dilution factor of the derivatization step. By these means, the LOQ might be lowered to 0.5 million cells in the case of K-0081 and 2.4 million cells in the case of K-0374. All biomass samples in this study had a measured prymnesin concentration between 640 µmol/mL and 2040 µmol/mL in the measurement solution. However, whether a peak is a peak is not only defined by the peak area, but also by the signal-to-noise ratio, which has to be assessed individually in each sample. 

Here, it should be pointed out that an HPLC-MS analysis of a given underivatized extract should be performed to confirm the presence and relative amount of prymnesins, especially in the case of not previously characterized *P. parvum* strains. To confirm the presence or absence and the absolute quantitation of one stereo-specific prymnesin analog, a standard with an exact concentration, which is currently not available, would be necessary. The derivatization procedure is quite unspecific and in case other primary amines are present in the extract and elute in the same retention time window as prymnesins, overestimation of the prymnesin content might occur. Additionally, a UV signal at 280 nm, visible at the same time as the underivatized or derivatised sample, is an indication of the presence of prymnesins. 

A semi-quantitative method utilizing the primary amine of prymnesins for quantitation has been previously developed by La Claire II et al. [17]. However, instead of fluorescence labeling the prymnesins directly from the biomass extract, they carried out a series of purification steps prior to quantitating the total amount of amines using a ninhydrin reaction and fluorescence detection. Furthermore, they provided no validation data for their method, so the limits of reliable quantitation and the apparent recovery of the method are unknown. The method presented here provides a simpler sample processing and validation of the data. 

### 2.3. Estimation of Prymnesin Levels in Biomass of Two P. parvum Strains during pH Limited Growth 

**Figure 2:**
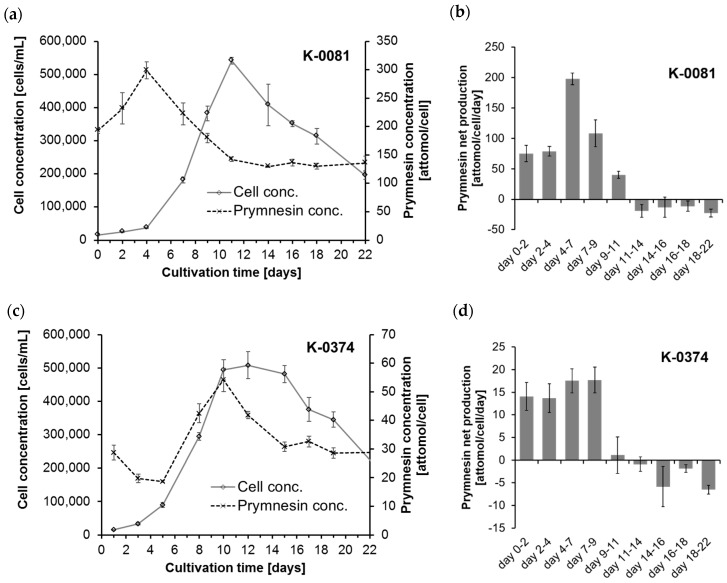
Growth and estimated prymnesin production of two *Prymnesium parvum* strains K-0081 and K-0374. The left graphs (**a**,**c**) show the algal cell concentrations and the estimated prymnesin content measured by fluorescence detection (*n* = 3). The graphs to the right (**b**,**d**) show the determined prymnesin net production in periods of 2–4 days during algal growth (*n* = 3). Note the difference in scaling of the Y-axis for the concentrations and prymnesin net production for the two strains. The cultures were pH and/or carbon limited, and the information on the pH, inorganic carbon speciation and carbon, nitrogen and phosphorous content in the biomass can be found in Figures S1 and S2 and Table S3.

The cellular amounts of prymnesins were determined for two B-type prymnesin producing strains (K-0081 and K-0374) at 10 different time points during 22 days of growth (0, 2, 4, 7, 9, 11, 14, 16, 18 and 22 days after inoculation). The cultures went through a whole growth cycle in the culturing period, including an initial lag phase, an exponential growth phase, a stationary and/or death phase caused by an elevated pH accompanied by carbon limitation (Figure 2). For the specific growth rates, see Table S2. Information on the pH, speciation of dissolved inorganic carbon (DIC), nitrogen (N) and phosphorous (P) in the cultures can be found in Figures S1 and S2 and Table S3. 

The highest cellular contents of prymnesins in strain K-0081 (299 ± 15 attomol per cell) were found in this experiment at a cell concentration of ~ 40,000 cells/mL (on day 4), while the highest cellular content of prymnesins in the K-0374 strain (54 ± 4 attomol per cell) was found at a cell concentration of ~500,000 cells/mL (on day 10) (Figure 2a,c). Thus, the maximum cellular contents of prymnesins in strain K-0081 were more than five times higher than the contents found in K-0374. The highest relative standard deviation was 12% for one of the early samplings of K-0081. The maximum net production rates for the two strains were 198 ± 9.5 attomol/cell/day (days 4–7) and 17.7 ± 2.5 attomol/cell/day (days 4–9) for strain K-0081 and K-0374, respectively (Figure 2b,d). Therefore, the prymnesin net production was ~5–10 times higher for K-0081 than for K-0374 during the early to late exponential growth phase. This is in accordance with earlier observations indicating that strain K-0081 was ~7 times more toxic towards the rainbow trout and more than 20 times more toxic towards other algae than strain K-0374 [24]. The molecular mass of B-type prymnesins range from 1655 Da to 2015 Da [13]; thus, the mass concentration of prymnesins depends greatly on the distribution of the different analogs that are present. The cellular content of prymnesins (by mass) was in the fg/cell range. Assuming that all prymnesins were of the lightest analog, the prymnesin content of the less toxic strain K-0374 was 31–90 fg/cell, while the more toxic strain (K-0081) contained 214–495 fg/cell.

The cellular prymnesin concentration changed during the growth phases. For both strains, a positive net production of prymnesins was found during the initial 11 days (see Figure 2b,d). This period was characterized by an exponential growth phase, increased cellular prymnesin concentrations and an increase in the seawater pH levels of up to 10.5 (see Figure 2 and Figure S1). The total amounts of inorganic carbon removed by the algae during the growth of the two cultures were on average ~12 mg C/L between day 2 and 11. This corresponds well with the amounts of carbon built into the algal biomass, which were 13.9 mg C/L at day 11 when both cultures were at their maximum concentrations. 

The elevated pH levels led to a shift of the carbon speciation towards less bicarbonate and more carbonate (see Figure S2). Due to the high pH on day 11, nearly all of the inorganic carbon was in the form of carbonate (ca. 99%), a carbon species that is unavailable to the algae, and thus very little inorganic carbon was left that the algae could utilize. Consequently, the algae entered the stationary phase (for K-0374) or death phase (for K-0081) at days 9–11, and the net toxin production became negative after day 11. From days 11–14 until day 22, the cellular prymnesin content was constant (see Figure 2a,c). The carbon limitation and/or the elevated pH hindered the algal cells from producing prymnesins. It is impossible with this experimental setup to determine if the algal growth and toxin production stopped due to the high pH (pH 10.5) or limitation of inorganic carbon (CO_2_ + HCO_3_^−^, ~0.100 µmol/L) at day 11. These two factors are known to limit growth among marine algae at these levels [25,26]. The algae were not nutrient limited, as they only removed ~8% of the added nitrate and phosphorous (see Table S3). 

Further investigations into the relative amounts of the individual prymnesin analogs were performed by HRMS-analysis (see Figure S3). Due to the lack of standards, the interpretation should be done with caution: On the one hand, the ionization efficiency might be different for the individual analogs, but on the other hand the matrix effects might vary between the growth stages, as well as between analogs eluting at different retention times. Although the ratios changed slightly over the growth curve, the general strain profiles stayed the same. In both investigated strains, the prymnesin aglycon backbone was the most pronounced, with a percentage of over 30%. In the case of B-type prymnesins, the in-source fragmentation of the glucose moieties-containing prymnesins to the aglycon backbone was only observed to a small extent. Therefore, a bias towards the aglycon is unlikely but still possible. Compared to strain K-0081, K-0374 showed a higher ratio of the prymnesin analog, with two incorporated chlorine atoms and one pentose unit (6–16% compared to 1–4%). In the case of strain K-0081, the B-type prymnesin with one incorporated chlorine-atom and one hexose unit was quite pronounced (24–34%), while this prymnesin analog was not detected in significant amounts in strain K-0374. Further investigations into the production of the different prymnesin analogs, as well as whether their production depends on the cultivation conditions, are necessary to better understand these differences. 

### 2.4. Extraction of Prymnesins from Cell Free Culture

The method development was based on material from the *P. parvum* strain K-0081. Five solid phase extraction (SPE) cartridges and two liquid-liquid extraction (LLE) methods were tested for the extraction of B-type prymnesins from a cell free supernatant at a salinity of 30. After an initial screening of the five SPE cartridges using mass spectrometric detection, the Isolute C8 cartridge was selected for further testing, along with the two LLE extraction methods (2-butanol (2-BuOH) and 2-BuOH:ethyl acetate (EtOAc), 1:1, *v*:*v*). The other SPE cartridges were excluded due to low recovery of the toxins (<20% recovery compared to the two LLE-methods). 

**Figure 3:**
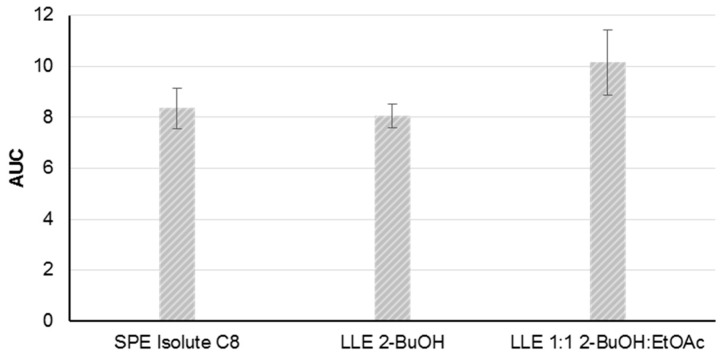
Comparison of the area under the curve (AUC) of the different extraction methods of prymnesins from cell free cultures. The extracted supernatant was from a high concentration (>500,000 cells/mL) algal culture of *Prymnesium parvum* K-0081, and the data were processed using AccQ-tagging and fluorescence detection. Solid phase extraction (SPE), as well as liquid/liquid extraction (LLE) with 2-butanol (2-BuOH) and a 1 + 1 mixture of 2-BuOH and ethyl acetate (EtOAc), showed a comparable AUC, but 2-BuOH showed the lowest standard deviation (*n* = 9, triplicates on three days).

The repeatability of the three extraction methods was assessed by the triplicate extraction of the supernatant from the same batch of a dense algal culture (>500,000 cells/mL) on three different days (*n* = 9). Following the sample preparation the prymnesin contents in the samples were assessed by fluorescence tagging and FLD-detection, as described in Section 4.4 and Section 4.5. Comparable peak areas and acceptable repeatabilities were achieved with relative standard deviations ranging from 6% to 13% (SPE C8: 9%, LLE 2-BuOH: 6%, and LLE 2-BuOH:EtOAc: 13%) (Figure 3). The LLE extraction with 2-BuOH was selected as the preferred choice due to the lower relative standard deviation. 

The validation of the LLE extraction method using 2-BuOH revealed that the apparent recovery depended on the salinity of the sample. In a culture media with a salinity of 9, an apparent recovery of only ca. 33% was obtained, while this value increased to approximately 68% in a culture media with a salinity of 30. However, when spiked samples with a salinity of originally 9 were supplemented with NaCl to obtain a salinity value of ~30 prior to the extraction, the same apparent recovery as for the medium with a salinity of 30 was achieved. Therefore, it is recommended that one increase the salinity content in the case of a low salinity medium in order to increase the recovery. However, the quantitation of prymnesins in the cell free samples was only possible over a limited range of high cell concentrations due to the low concentration of prymnesins in water (low pmol/mL range, low ng/mL range).

### 2.5. Fractions of Prymnesins in Water and Algal Biomass During Growth Phases

The effect of prymnesins on fish is believed to be caused by prymnesins that have been secreted or leaked into the surrounding water [27]. Therefore, the fraction of prymnesins in whole cell cultures, culture filtrates and culture supernatants were studied for one *P. parvum* strain (K-0081) at five different time points (5, 10, 12, 17 and 21 days after inoculation). The samples were processed according to the LLE (2-BuOH) extraction procedure described in Section 4.8.2, and the peak areas obtained in the cell free samples (filtrate or supernatant) were compared to those of the whole cell culture. A typical FLD-chromatogram of an algal extract and a magnification of the prymnesin peak can be found in Figure S5. Since the FLD-method only allowed the quantitation of prymnesins in the cell-free samples at days 10 and 12, mass spectrometric detection was primarily applied. HPLC-HRMS offered lower detection limits, but only a relative comparison without an absolute quantitation due to the lack of standards. Figure S6 shows a typical HRMS-chromatogram. 

**Figure 4:**
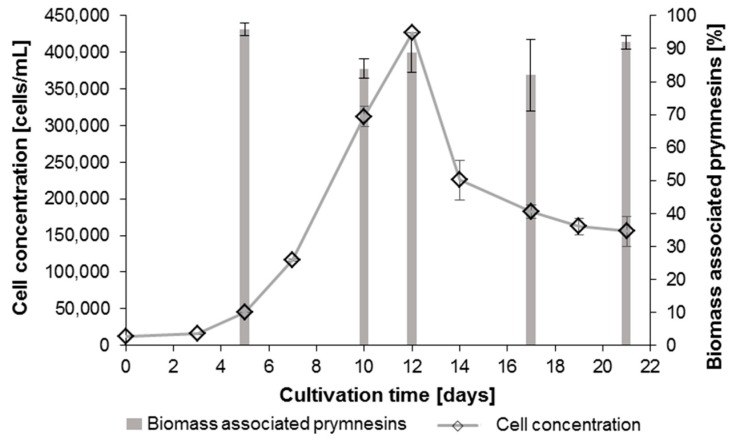
The growth curve of *P. parvum* strain K-0081 and determination of the percentage of prymnesins associated with the biomass by a relative comparison of the prymnesin content in whole cultures and cell-free filtrates by liquid chromatography-mass spectrometry (*n* = 3). The pH in the cultures became quite high, pH > 10, and the cultures became inorganic carbon limited; for details see Figure S4.

The main fraction of prymnesins was in the algal biomass, regardless of the used separation technique, and the percentage of prymnesins in the water or bound in the algal biomass was quite constant. Based on the comparison of the filtrate and whole cell culture, the FLD-data showed that 82 ± 1% of the prymnesins were associated with the biomass in the exponential phase (day 10) at 312,000 cells/mL, and a similar amount of prymnesins was found at the peak cell concentration of 427,000 cells/mL (day 12) (see Table S4 for details). Importantly, the ratios were similar with the HPLC-HRMS method (Figure 4), and the overall percentage of prymnesins associated with the biomass was 89% ± 7% (see Table S5 for details). No statistically significant difference was found in biomass related prymnesins during 21 days of growth (one-way analysis of variance (ANOVA), *p* = 0.08). The algal growth became limited by an elevated pH (as high as 10.5) in the same manner as the first growth experiment (see Figure S4 for the pH curve). Plenty of nutrients (N and P) were left at the peak of the algal biomass (see Table S3). 

Interestingly, the cellular prymnesin content remained constant even though the *P. parvum* cells started to die out. This indicates a very fast degradation of prymnesins during this phase, which is subject to further investigations. Furthermore, this is in contrast to the properties of other phycotoxins e.g., paralytic shellfish poisoning (PSP), diarrhetic shellfish poisoning (DSP), and domoic acid, which seem more stable. They have been found to accumulate in the cells when expressed as cellular content during stationary and death phase [28,29], as well as extracellularly in the water and/or detritus. 

Comparing the supernatant with the whole cell culture revealed a lower percentage of prymnesins in the biomass (54–75%), indicating that the mode of sampling has an influence on the prymnesin concentration in the cell free samples. It seems that centrifugation leads more easily to the disruption of the cells and hence to higher amounts of prymnesins in the water. Xu et al. [30] showed that centrifugal stress has an influence on the cell disruption and glycerol leakage of *Dunaliella salina*, a cell wall lacking microalgae. Detailed data for all of the time points can be found in Table S4 for the FLD-method, and in Table S5 for the HRMS-method.

The observed findings are contrary to the findings previously reported, where the majority of prymnesins were found in the supernatant [17]. Possible reasons for this could be that different culture conditions influence the ratio of prymnesins in the biomass and cell free samples or that the applied centrifugation step caused the disruption of the cells and release of the prymnesins into the water. Furthermore, La Claire et al. [17] did not compare whole cell cultures and cell free supernatant, but assessed the prymnesins in the biomass and the supernatant independently, which might be associated with different recoveries. Taken together, further studies are needed to investigate how external factors affect the ratio of prymnesins in water and biomass. 

## 3. Conclusions

Here, methods for assessing the amount of prymnesins in water and biomass using fluorescence tagging of the primary amine and fumonisins as external calibrants were presented. A part of future research should be considerations into the improvement of the apparent recoveries (33% for cultures with a salinity of 9, 68% for those at a salinity of 30 and 58% for the biomass analysis). In particular, a special focus could be to increase the recovery of the final evaporation/reconstitution step currently responsible for ca. 75% of the losses. HRMS-analyses were applied to confirm the presence of prymnesins, to provide relative comparisons, and to study the different prymnesin analogs that were produced. Due to the lack of standards, HRMS cannot be used for quantitative purposes. The currently available extraction and quantitation procedure of prymnesins from cell free cultures is not sensitive enough for monitoring purposes due to the low prymnesin concentrations in water. This is greatly influenced by the fact that the vast majority of prymnesins are associated with the biomass (89% ± 7%) and that only a small extent is released into the water. It was further shown that the mode of sampling influences the amount of prymnesins in water and that filtration causes less prymnesin release into the water than centrifugation does. The amount of prymnesins in the biomass seems to depend on the one hand on the growth stage (ca. a factor two difference between the base and peak level prymnesin content/cell) and on the other hand on the specific strain (K-0081 showed a higher amount of prymnesins per cell than strain K-0374). Therefore, care has to be taken when designing future experiments with unknown strains, since the required amount of sampled cells seems to be strain specific. Given their similar chromatographic properties, the presented method should be easily transferable to the quantitation of prymnesins belonging to the other two toxin groups (A- and C-type). The presented methods will allow the exploration of how external factors influence the overall prymnesin production and whether the relative ratios of the different prymnesin analogs are influenced. 

## 4. Materials and Methods 

### 4.1. Reagents, Standards and SPE Cartridges 

Solvents used for the sample preparation and HPLC-FLD analysis were all HPLC-grade, while solvents used for the HPLC-HRMS analysis were MS-grade. Acetonitrile (ACN), ammonium formate, formic acid, ethyl acetate, MeOH, and trifluoroacetic acid were all purchased from Sigma-Aldrich (Schnelldorf, Germany), while 2-BuOH was purchased from Fisher Scientific (Waltham, MA, USA). Water was purified by reverse osmosis and a subsequent MilliQ-system (Millipore, Bedford, MA, USA). Fluorescence derivatization was achieved using AccQ-Fluor reagent WAT052880 (Waters, Milford, MA, USA). External calibration was carried out using a fumonisin B_1_ (49.9 µg/mL) and fumonisin B_2_ (50.6 µg/mL) mixture in H_2_O:ACN (1:1, *v*/*v*), purchased from Romer Labs (Tulln, Austria). Four different Isolute SPE cartridges (C18 (MFC) (3 mL, 500 mg), C8 (3 mL, 500 mg), C2 (3 mL, 500 mg) and Isolute Myco (3 mL, 60 mg)) were purchased from Biotage (Uppsala, Sweden), while Strata-X (3 mL, 30 mg) SPE cartridges were purchased from Phenomenex (Torrance, CA, USA). 

### 4.2. Algal Cultures and Growth Experiments

The *P. parvum* strains K-0081 and K-0374 were both acquired from the Scandinavian Culture Collection for Algae and Protozoa (University of Copenhagen, Copenhagen, Denmark) now merged into NIVA Culture Collection of Algae (NIVA-CCA) (University of Oslo, Oslo, Norway). The cultures were all grown in pasteurized (95 °C for 90 min) seawater-based F/2-media [31]. The seawater was sampled below the pycnocline off the coast of Elsinore (Denmark) and had a salinity of ca. 30. When needed, salinity 9 medium was prepared by diluting salinity 30 seawater with demineralized water before adding F/2 nutrients. NaHCO_3_ was added (28 mL 0.5 M to 10 L medium) in order to compensate for the lower carbon concentration in demineralized water. During cultivation, the temperature was kept at 15 °C, and an irradiation of 150–200 µmol photons/m^2^/s was maintained with a light-dark cycle of 14:10 h. 

The cultures used for the method development were previously acclimatized and grown at salinity 30 in 5 or 10 L bottles with gentle aeration. Two growth experiments were conducted, and both were performed with salinity 9 F/2-media in 10 L air sealed glass bottles with no aeration in order to pH limit the cultures. In the first growth experiment (quantitation of prymnesins in biomass of two *P. parvum* strains during pH limited growth), two sets of triplicate bottles were inoculated with the *P. parvum* strains K-0081 and K-0374, respectively. The cultures used for the inoculation were in their exponential growth phase and were added to reach an initial concentration of 5000 cells/mL. The cultures were grown for 22 days. In the second growth experiment (determination of the ratio of prymnesins in water and biomass), 10 L bottles were inoculated in triplicate with strain K-0081 to have an initial concentration of 10,000 cells/mL and were then grown for 21 days. 

### 4.3. Determination of Biological Parameters and Prymnesin Production

In both growth experiments, the samples for the determination of the algal cell concentration, pH and prymnesin concentration were taken every 2–5 days during the growth period. The pH-values were measured with a WTW 3210 pH meter (Weilheim, Germany), and the samples were fixed with acidic Lugols (2% final concentration) for the cell counts and counted on an inverted microscope (Olympus CKX53, London, United Kingdom). In the first experiment, the samples were taken at days 2, 11 and 22 for the measurements of inorganic carbon (IC) in the filtered samples. The filters (Whatman GF/F, Sigma Aldrich, St. Louis, MI, USA) were heat treated (450 °C for at least 4 hours) prior to use. The samples were analyzed on a Shimadzu TOC-L analyzer (Shimadzu Corporation, Kyoto, Japan), and the speciation of inorganic carbon into carbon dioxide (CO_2_), bicarbonate (HCO_3_^−^) and carbonate (CO_3_^2−^) was calculated in the Excel CO2Sys MACRO ([32] created by Dr. D. Pierrot using the code developed by [33], using the set constants K1, K2 from [34], and refit by [35]). The calculations of the carbon in the biomass are based on the equation from [36], and the C:N and C:P ratios from [37] were used to calculate nitrogen and phosphorous in the algal biomass (see Figure S2 for the speciation of inorganic carbon and Table S3 for the content of carbon, nitrogen and phosphorous in the biomass). See Section 4.7, Section 4.8 and Section 4.9 for the toxin sample preparation for the two growth experiments. 

The prymnesin production was determined for periods of 2–4 days by multiplying the algal growth rate (K) in the specific period with the average prymnesin concentration for the same period (see Equations (1) and (2)). N_0_ and N_1_ are the number of cells at the time points t_0_ and t_1_, and µ is the specific growth rate.

(1) $$K=\frac{\mu }{\ln\left(2\right)}$$

(2) $$\mu =\frac{ln\left(\frac{N_{1}}{N_{0}}\right)}{t_{1}-t_{0}}$$

### 4.4. Quantitation of Prymnesins

The quantitation was achieved by the fluorescence tagging of the primary amine in prymnesins using a mixture of the mycotoxins fumonisin B_1_ and B_2_ as external calibrants. The fluorescence tagging was carried out with the AccQ-Fluor Reagent Kit from Waters (Milford, USA). The tagging of both the samples and calibration standards was carried out according to the manufacturer’s instructions for derivatized samples. A sample aliquot (20 µL) was diluted in 60 µL borate buffer and vortexed prior to adding 20 µL of the AccQ-Fluor reagent dissolved in ACN. The mixtures were vortexed and allowed to stand for 1 min, before heating them for 10 min at 55 °C in a heating block. In the case of the determination of the ratio of prymnesins in the water and biomass, the ratio during the derivatization procedure was changed (60 µL sample, 20 µL borate buffer and 20 µL AccQ-Fluor reagent) due to the low amounts of prymnesins present in the sample. 

The following concentration levels prior to the derivatization were used for fumonisin B_1_: 49.9 µg/mL, 15.0 µg/mL, 9.98 µg/mL, 4.99 µg/mL, 1.50 µg/mL, 0.499 µg/mL, and 0.150 µg/mL; and 50.6 µg/mL, 15.2 µg/mL, 10.1 µg/mL, 5.06 µg/mL, 1.52 µg/mL, 0.506 µg/mL, and 0.152 µg/mL for fumonisin B_2_. The lowest level was just for indicative purposes because the resulting peak areas were below one, which is the minimum requirement of the software. The concentration of prymnesins in the measurement solution was calculated by averaging the concentration calculated based on each of the two linear non-weighted calibration curves from fumonisin B_1_ and B_2_. Since the peak areas of the two fumonisins were not exactly the same (ratio fumonisin B_1_:fumonisin B_2_ was ca. 0.75–0.80), taking the average was considered to be more appropriate for an approximate quantitation. The correction factors for the dilution or concentration steps and apparent recoveries were applied. 

### 4.5. HPLC-FLD 

The chromatographic separation was performed on an 1100 HPLC system (Agilent Technologies, Waldbronn, Germany) using a 100 × 2.1 mm, 3 µm Luna C18(2) column (Phenomenex, Aschaffenburg, Germany) at 40 °C and at a flow rate of 0.400 mL/min. A linear water-ACN gradient, containing 50 ppm trifluoroacetic acid, was applied starting with 20% ACN for 1 min, followed by a linear increase to 100% ACN over 7 min. Then, the column was flushed with 100% ACN for 2 min before returning to the start conditions. The samples were maintained at room temperature in the autosampler, and in general 5 µL (fumonisin standards) or 10 µL (samples) were injected. In some cases, 20 µL were used in addition to increase the signal. The fluorescence data were acquired on an Agilent 1100 fluorescence detector (Agilent Technologies) operating in λ excitation 250 nm and emission 395 nm. The data were evaluated using ChemStation (Rev. B01.01) and Microsoft Excel 2016. 

### 4.6. HPLC-DAD-HRMS

For HPLC-DAD-HRMS analyses the underivatized extracts after reconstitution in MeOH:H_2_O (90:10, *v*/*v*) were used. Separation was performed on an Ultimate 3000 UHPLC system (Dionex, Sunnyvale, USA) including a diode array detection (DAD) system using a 100 × 2 mm, 2.6 µm Kinetex C18 column (Phenomenex, Aschaffenburg, Germany) at 40 °C and a flow rate of 0.400 mL/min. A linear water-ACN gradient, containing 20 mM formic acid, was applied from 10% to 100% ACN in 10 min and maintained for 2 min before returning to the start conditions. The samples were maintained at 8 °C in the autosampler and 5 µL were injected for each analysis. 

Mass spectrometric data were acquired on a MaXis HD QTOF-MS (Bruker Daltonik, Bremen, Germany) equipped with an electrospray ionization source. The mass spectrometer was operated in positive mode with a capillary voltage of 4500 V recording data in a scan range from *m*/*z* 300 to 2500 at a rate of 2 scans/sec. The drying gas flowrate was set to 10.0 L/min, the temperature to 200 °C and the nebulizer pressure was 1.8 bar (180 kPa). To accommodate larger ions, the following settings were used for the collision cell: transfer time 100 µs, collision cell RF 1500 Vpp, and prepulse storage 10 µs. The mass spectrometer was calibrated using sodium formate. Prymnesins were identified by their accurate mass (mass error < 5 ppm), their distinctive isotopic patterns due to the presence of at least one chlorine atom and the in-source fragmentation of the sugar-moieties to the aglycon. An overview over the currently known B-type prymnesins is provided in Table S1. In general, the double charged ions were more pronounced than the single charged ones. Further confirmation of prymnesins, in previously not characterized strains, is necessary using tandem mass spectrometry. Data were evaluated with Bruker Compass DataAnalysis 4.2 (Build 383.1) and Microsoft Excel 2016. 

### 4.7. Sample Preparation from Biomass

The sampling was carried out either by centrifugation (4248 g, 15 min) or by gentle suction filtration, using one or multiple glass microfiber filters (Whatman GF/C 1.2 µm, diameter 47 mm, Sigma Aldrich, St. Louis, MO, USA), depending on the blocking. Both the funnel and filters were autoclaved prior to use. The number of cells needed for the sampling was determined in a pre-experiment. For the sampling of strain K-0374, 60 million cells were required, whereas 9 million cells were sufficient for K-0081 due to the different amount of prymnesins produced by the two algal strains under the given conditions. Prior to each sampling, the cultures were counted using a microscope, and the required volume was calculated. After sampling, the samples were stored at -80 °C until further processing. 

The extraction was performed by the addition of 20 mL MeOH to the biomass. Thereafter, the samples were briefly vortexed and sonicated for 30 min, followed by a final centrifugation step (4248 g, 4 °C, 15 min). The MeOH-extracts were decanted into new polypropylene tubes, and the procedure was repeated once more. The combined MeOH-extracts (40 mL) were dried under N_2_ at 35 °C, reconstituted in 1 mL MeOH:H_2_O (90:10, *v*/*v*) and transferred into glass HPLC-vials. In the case of the biomass in the filter samples, the reconstituted samples were transferred first to 1.5 mL Eppendorf tubes and centrifuged (12,100 g, room temperature, 3 min) to separate residues from the glass fiber filters. Thereafter, the residue free extracts were transferred to glass HPLC-vials. 

### 4.8. Sample Preparation from Water Samples

For the method development, the cells were removed by centrifugation (4248 g, 4 °C, 15 min) prior to any sample preparation to obtain cell free water. The samples were stored at −80 °C until further processing. 

#### 4.8.1. SPE

For the initial screening, the five different columns (Isolute C18 (MFC), Isolute C8, Isolute C2, Isolute Myco and Strata-X) were first washed with 5 mL MeOH and then conditioned with 5 mL H_2_O. The samples (40 mL cell free water) were loaded, and the columns were washed with 5 mL H_2_O. Thereafter, the columns were eluted stepwise with 10%-increments from 20% to 80% MeOH in water, followed by MeOH:H_2_O:isopropanol (1:1:1, *v*/*v*/*v*) and MeOH:H_2_O:2-BuOH (1:1:1, *v*/*v*/*v*) (5 mL each). Elution tests were performed with or without the addition of 1% formic acid to all of the solvents. The samples were collected in 15 mL polypropylene tubes, dried using an N_2_ evaporation unit at 35 °C and reconstituted in 1 mL MeOH. 

For the final SPE method, the Isolute C8 column was selected, the columns were washed with MeOH (5 mL), conditioned (5 mL H_2_O) and then loaded with the sample (40 mL). The columns were washed with 5 mL H_2_O and with 5 mL MeOH:H_2_O (40:60, *v*/*v*), and the elution was performed with 5 mL MeOH:H_2_O (90:10, *v*/*v*). All three washing and elution solvents contained 1% formic acid. The samples were dried using an N_2_ evaporation unit at 35 °C and reconstituted in 1 mL MeOH:H_2_O (90:10, *v*/*v*), since an increased recovery was observed when compared to 100% MeOH. 

#### 4.8.2. LLE

For the liquid-liquid extractions, either pure 2-BuOH or a 1 + 1 mixture of EtOAc and 2-BuOH were used. The extractions were carried out in a separation funnel by first extracting 40 mL of the sample with 20 mL of the chosen solvent, followed by extracting twice with 10 mL. The three organic phases were combined and washed twice with 20 mL H_2_O. The resulting extracts were dried under nitrogen at 35 °C and reconstituted in 1 mL MeOH in the pre-experiments and in 1 mL MeOH:H_2_O (90:10, *v*/*v*) in the comparison with the Isolute C8-column. 

### 4.9. Determination of the Ratio of Prymnesins in Water and Biomass

The algal cultures of strain *P. parvum* K-0081 were cultivated as described above (Section 4.2) in F/2-media with a salinity of 9 without aeration in triplicate. In this experiment, the used seawater was passed through a 0.22 µm white plain filter using a Millipore filtration system prior to autoclaving. The whole cell culture and cell free samples were taken at different time points over the growth curve (day 5, 10, 12, 17, and 21). For the whole cell culture, 40 mL aliquots were transferred to polypropylene tubes. The cell-free samples were either obtained by the gentle suction filtration (Whatman GF/C 1.2 µm, diameter 47 mm, Sigma Aldrich, St. Louis, USA) of the 40 mL whole cell culture (=filtrate) or by the centrifugation (4,200 g for 15 min at room temperature) of the 40 mL whole cell culture (=supernatant). The filtrates or supernatants were transferred to polypropylene tubes. All three sample sets (whole cell culture, filtrate and supernatant) were extracted using the LLE-procedure with pure 2-BuOH, as described above (Section 4.8.2). The measurements were performed with both the HPLC-FLD (Section 4.5) and the HPLC-HRMS (Section 4.6) methods.

The percentages of prymnesins in water were calculated as the ratio of the peak area obtained from the filtrate or supernatant and the peak area of the whole cell culture, multiplied with 100. To obtain the percentage of the biomass-associated prymnesins, this value was subtracted from 100. In the case of the HPLC-FLD analysis, the peak area under the fluorescence curve was used for calculation. For the HPLC-HRMS analysis, the sum of the extracted ion chromatograms of single and double charged protonated ion species (±*m*/*z* 0.02) of previously identified B-type prymnesins was taken into consideration [13]. 

### 4.10. Validation

To assess the apparent recovery of prymnesins, the MeOH-extract of a *P. parvum* K-0081 culture was used for spiking purposes, and assessments were performed in triplicate. The apparent recovery of the LLE-method was determined by spiking F/2-media with a salinity of 9 and 30 in triplicate with a known amount of extract and carrying out the extraction as described in Section 4.8.2, using 2-BuOH as the extraction solvent. Furthermore, the spiked F/2-media with a salinity of 9 was adjusted to a salinity of ca. 30 by the addition of NaCl to assess whether the apparent recovery could be improved. In the case of the determination of prymnesins in the biomass, a known amount of the prymnesin extract was added to a filter and was allowed to dry before carrying out the extraction procedure, as described in Section 4.7. Both the HPLC-FLD (Section 4.5) and HPLC-DAD-HRMS (Section 4.6) analyses were carried out. In all of the cases, the obtained peak areas of the samples that were spiked before the extraction were compared with those of the references samples that were simply diluted to the equivalent level (*n* = 3). 

Furthermore, the influence of the drying down/reconstitution step was assessed by adding a prymnesin extract to 40 mL MeOH in a polypropylene tube in triplicate. The samples were tried down, reconstituted in 1 mL MeOH:H_2_O (90:10, *v*/*v*) and also compared to the reference sample. The theoretic limits of the methods were determined using the calibration curve method, as described in [23]. An extract of K-0081 was diluted, and the relative standard deviation of the peak area of ten replicates was calculated. This value was multiplied with three and ten, respectively, and the calibration curves of fumonisin B_1_ and B_2_ were used to obtain the LOD and LOQ.

## Supplementary Materials

The following are available online at https://www.mdpi.com/2072-6651/11/5/251/s1, Figure S1: Seawater pH as a function of the growth of the two *Prymnesium parvum* strains during the first growth experiment (Quantitation of prymnesins in the biomass of two *P. parvum* strains during pH limited growth), (*n* = 3); Figure S2: Total inorganic carbon (IC) and speciation of inorganic carbon into carbon dioxide (CO_2_), bicarbonate (HCO^3−^) and carbonate (CO_3_^2−^) measured three times (days 2, 11 and 22) during the first growth experiment (Quantitation of prymnesins in the biomass of two *P. parvum* strains during pH limited growth), (*n* = 3); Figure S3: Relative ratios of the individual prymnesin peak areas as a function of the sum of all prymnesin peak areas present in the respective sample, (*n* = 3); Figure S4: Seawater pH as a function of the growth of *Prymnesium parvum* K-0081 during the second growth experiment, (Determination of the ratio of prymnesins in the water and in the biomass), (*n* = 3); Figure S5: Typical fluorescence detection (FLD) chromatogram of a whole cell culture of *Prymnesium parvum* strain K-0081 containing B-type prymnesins after the liquid-liquid extraction and derivatization with AccQ-Fluor reagent and magnification of the prymnesin peak (230 nmol/L); Figure S6: Typical high resolution mass spectrometric (HRMS) chromatograms of a whole cell culture containing B-type prymnesins; Table S1: Overview of currently known B-type prymnesins; Table S2: Specific growth rates (µ) calculated for periods of 2–4 days during the first algal growth experiment (Quantitation of prymnesins in the biomass of two *P. parvum* strains during pH limited growth). Table S3: Content of carbon (C), nitrogen (N) and phosphorus (P) in the algal biomass calculated at day 11 of the first growth experiment (Quantitation of prymnesins in the biomass of two *P. parvum* strains during pH limited growth) where the algae cultures achieved the maximum biomass, (*n* = 3); Table S4: Percentage of biomass-associated prymnesin content based on the comparison of the whole cell culture with either the filtrate or supernatant, determined by the liquid chromatographic (LC)—fluorescence detection (FLD) method, (*n* = 3); Table S5: Percentage of the biomass-associated prymnesin content based on the comparison of the whole cell culture with either the filtrate or supernatant determined by the liquid chromatographic (LC)—high resolution mass spectrometric (HRMS) detection method, (*n* = 3).
