# Supplementary material for: Development of an Indirect Quantitation Method to Assess Ichthyotoxic B-Type Prymnesins from Prymnesium parvum
Source: Toxins (Basel). 2019 May 4;11(5):251. doi: 10.3390/toxins11050251 (PMC6563205; doi:10.3390/toxins11050251)
Supplement: Supplementary file 1 [file toxins-11-00251-s001.pdf]

Supplementary Materials: Development of an Indirect Quantitation Method to Assess Ichthyotoxic B-Type Prymnesins from *Prymnesium parvum*

Daniel Killerup Svenssen, Sofie Bjørnholt Binzer, Nikola Medić, Per Juel Hansen, Thomas Ostenfeld Larsen and Elisabeth Varga

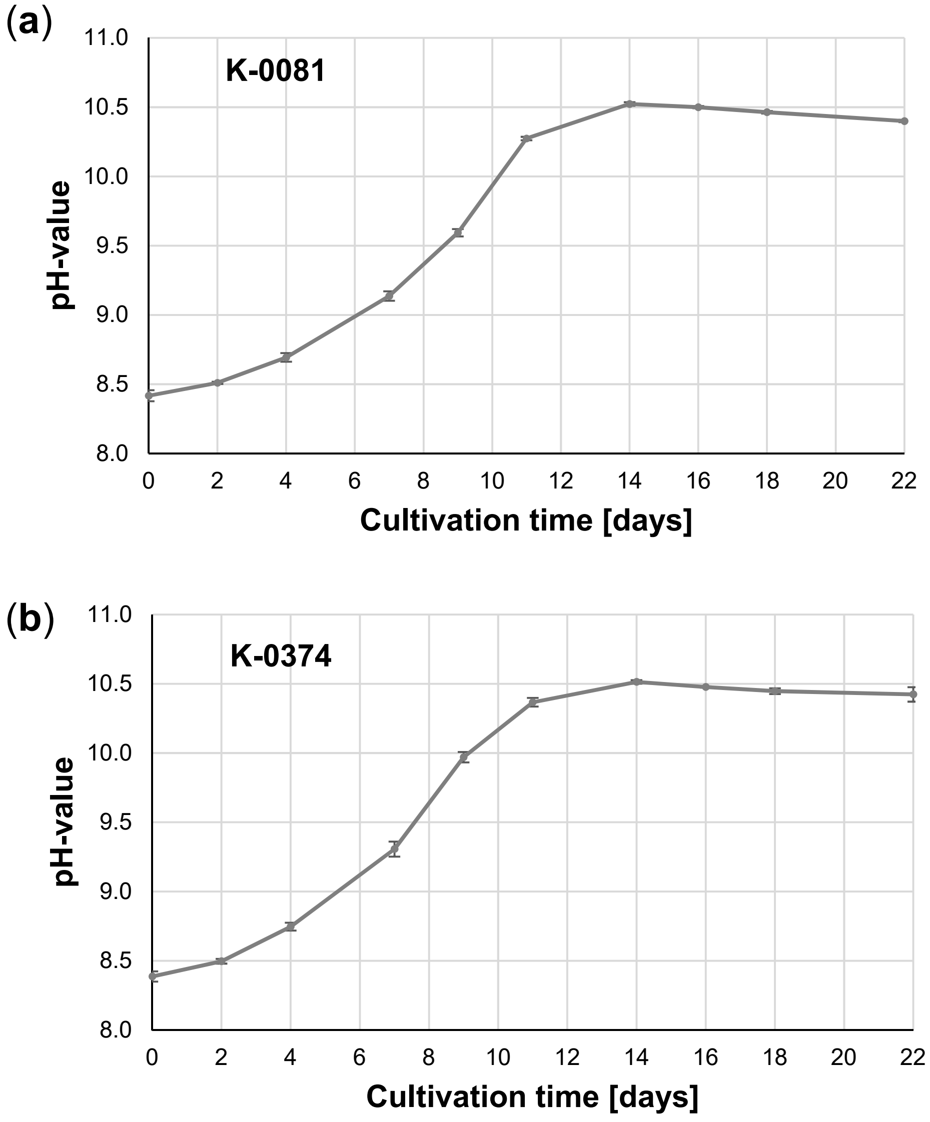

Figure S1. Seawater pH as a function of the growth of the two *Prymnesium parvum* strains during the first growth experiment (Quantitation of prymnesins in the biomass of two *P. parvum* strains during pH limited growth), (n = 3) (**a**) strain K-0081, (**b**) strain K-0374.

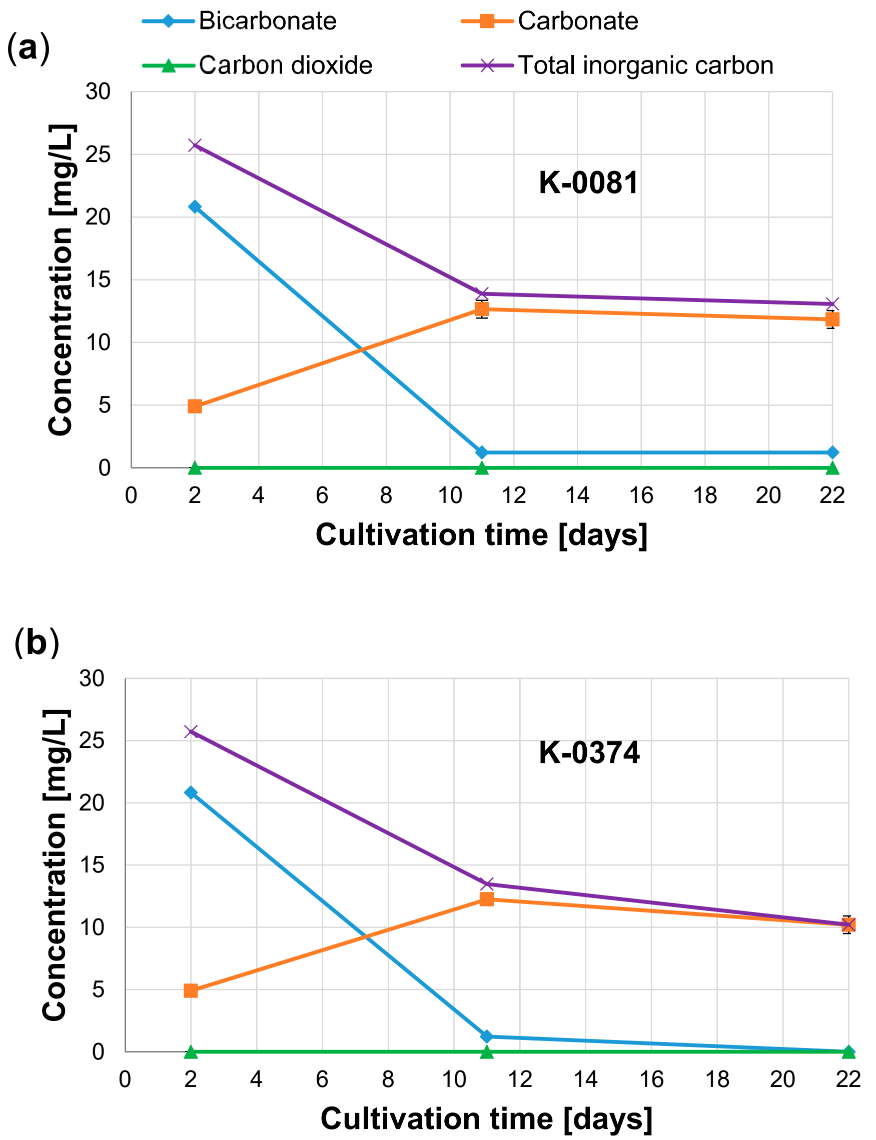

Figure S2. Total inorganic carbon (IC) and speciation of inorganic carbon into carbon dioxide (CO_2), bicarbonate (HCO_3^-) and carbonate (CO_3^{2-}) measured three times (days 2, 11 and 22) during the first growth experiment (Quantitation of prymnesins in the biomass of two *P. parvum* strains during pH limited growth), (*n* = 3). Triplicate samples from the two strains (K-0081 (**a**) and K-0374 (**b**)) were analyzed for inorganic carbon content on a Shimadzu TOC-L CSN analyzer (Shimadzu Corporation, Kyoto, Japan). The speciation of inorganic carbon into CO_2, HCO_3^- and CO_3^{2-} were calculated in the excel CO2Sys MACRO (created by [1] using the code developed by [2]) using the set constants K1, K2 from [3] refit by [4].

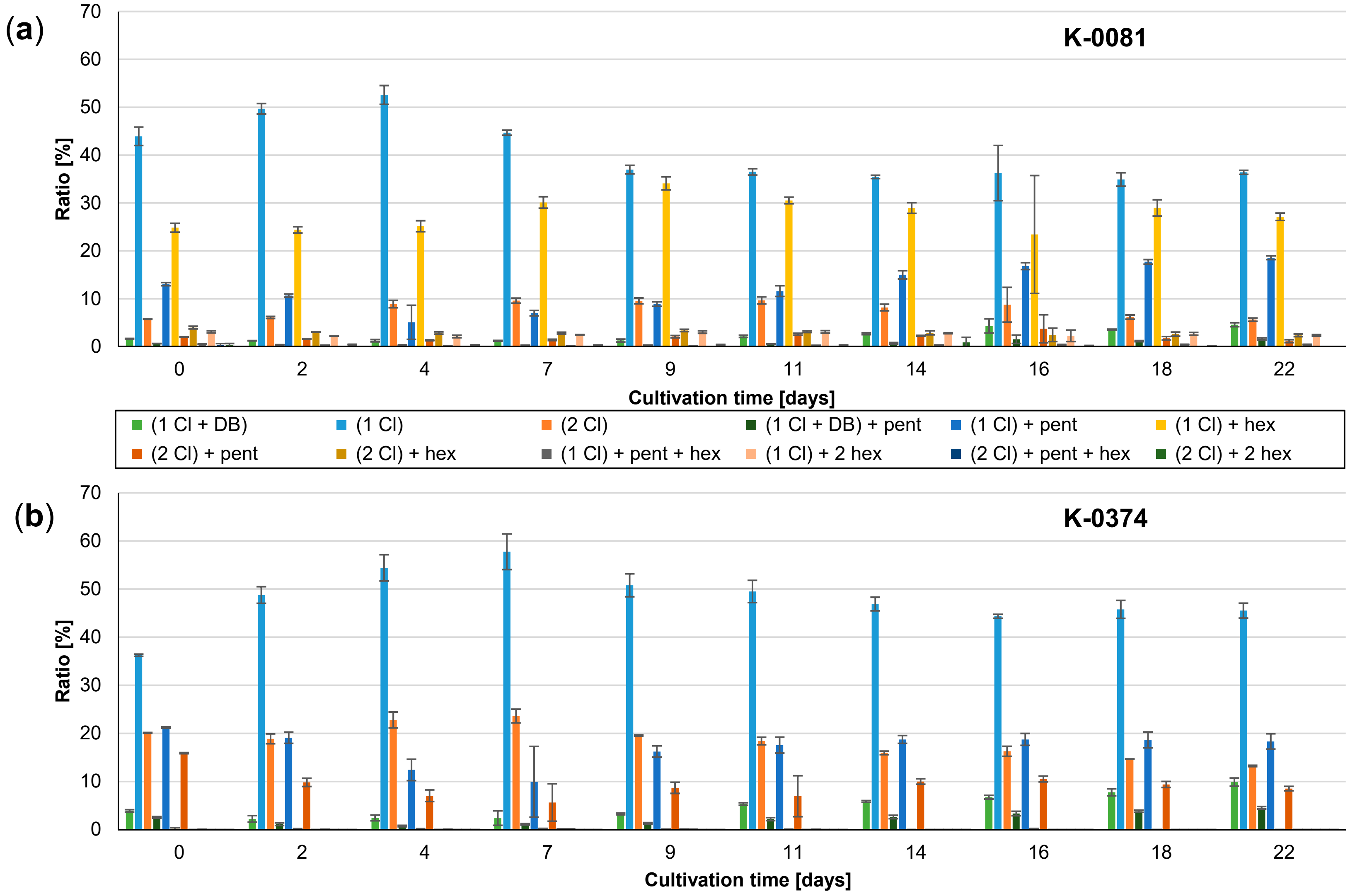

Figure S3. Relative ratios of the individual prymnesin peak areas as a function of the sum of all prymnesin peak areas present in the respective sample, (n = 3) (**a**) strain K-0081, (**b**) strain K-0374. Abbreviations: (1 Cl + DB) – prymnesin B-type backbone with one incorporated chlorine-atom and one additional double bond; (1 Cl) – prymnesin B-type backbone with one incorporated chlorine-atom; (2 Cl) – prymnesin B-type backbone with two incorporated chlorine atoms; pent – pentose conjugate; hex – hexose conjugate

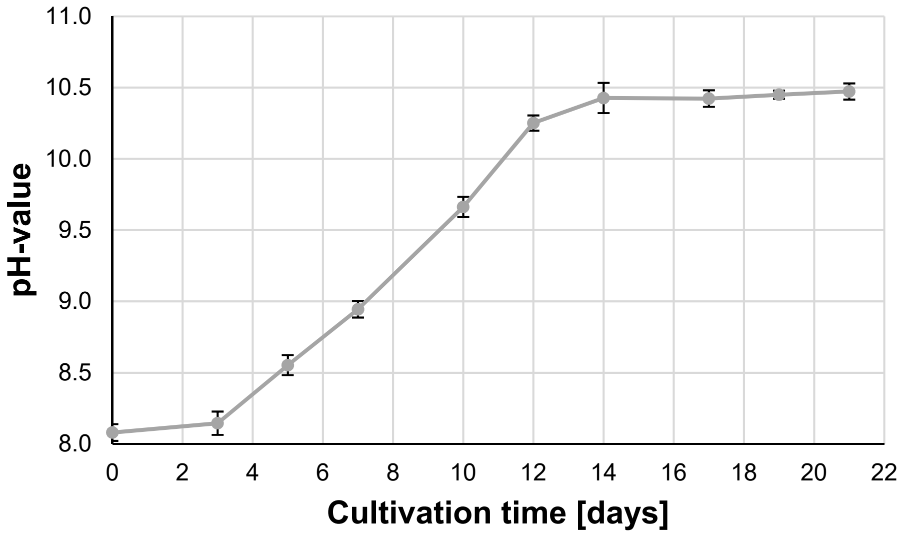

Figure S4. Seawater pH as a function of the growth of *Prymnesium parvum* K-0081 during the second growth experiment, (Determination of the ratio of prymnesins in the water and in the biomass), (n = 3).

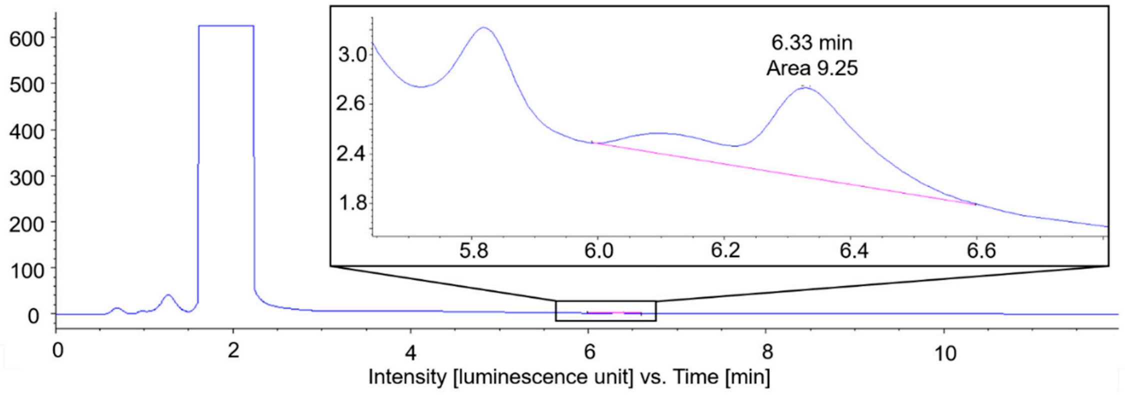

Figure S5. Typical fluorescence detection (FLD) chromatogram of a whole cell culture of *Prymnesium parvum* strain K-0081 containing B-type prymnesins after the liquid-liquid extraction and derivatization with AccQ-Fluor reagent and magnification of the prymnesin peak (230 nmol/L).

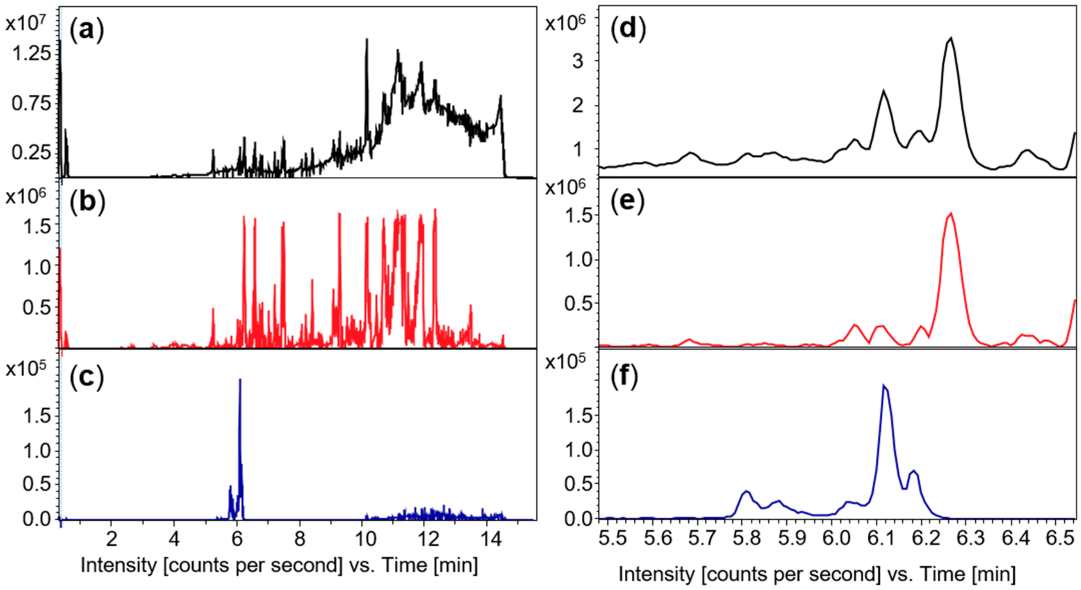

Figure S6. Typical high resolution mass spectrometric (HRMS) chromatograms of a whole cell culture containing B-type prymnesins (**a**) total ion chromatogram (TIC), (**b**) base peak chromatogram (BPC), (**c**) sum of extracted ion chromatograms of single and double charged protonated ion species ± *m*/*z* 0.02 of previously identified B-type prymnesins. (**d**), (**e**) and (**f**) are the same chromatogram types, but only displaying the retention time window between 5.5 and 6.5 min.

Table 1. Overview of currently known B-type prymnesins.

Proposed systematic name	Proposed sum formula	exact masses		
		$[M+H]^+$	$[M+2H]^{+2}$	$[M+Na+H]^{+2}$
PRM-B (1 Cl + DB)	$C_{85}H_{120}ClNO_{29}$	1654.7707	827.8890	838.8800
PRM-B (1 Cl + DB) + pentose	$C_{90}H_{128}ClNO_{33}$	1786.8130	893.9101	904.9011
PRM-B (1 Cl)	$C_{85}H_{122}ClNO_{29}$	1656.7864	828.8968	839.8878
PRM-B (1 Cl) + pentose ~ prymnesin-B2	$C_{90}H_{130}ClNO_{33}$	1788.8286	894.9180	905.9089
PRM-B (1 Cl) + hexose ~ prymnesin-B1	$C_{91}H_{132}ClNO_{34}$	1818.8392	909.9232	920.9142
PRM-B (1 Cl) + pentose + hexose	$C_{96}H_{140}ClNO_{38}$	1950.8815	975.9444	986.9353
PRM-B (1 Cl) + 2 hexose	$C_{97}H_{142}ClNO_{39}$	1980.8920	990.9497	1001.9406
PRM-B (2 Cl)	$C_{85}H_{121}Cl_2NO_{29}$	1690.7474	845.8773	856.8683
PRM-B (2 Cl) + pentose	$C_{90}H_{129}Cl_2NO_{33}$	1822.7897	911.8985	922.8894
PRM-B (2 Cl) + hexose	$C_{91}H_{131}Cl_2NO_{34}$	1852.8002	926.9038	937.8947
PRM-B (2 Cl) + pentose + hexose	$C_{96}H_{139}Cl_2NO_{38}$	1984.8425	992.9249	1003.9159
PRM-B (2 Cl) + 2 hexose	$C_{97}H_{141}Cl_2NO_{39}$	2014.8531	1007.9302	1018.9211

Prepared according to [5-7]. PRM-B: B-type prymnesin with 85 carbon-atoms in the backbone; Cl: number of chlorine-atoms in the proposed compounds; + DB: additional double bond; + pentose: pentose-conjugate attached; + hexose: hexose-conjugate attached; ~: sum formula equals to the previously reported prymnesin.

Table S2. Specific growth rates (μ) calculated for periods of 2-4 days during the first algal growth experiment (Quantitation of prymnesins in the biomass of two *P. parvum* strains during pH limited growth). Growth rates were calculated according to equation 2 in materials and methods, (mean ± SD, n = 3).

Growth rate (μ)		
**Time**	**K-0081**	**K-0374**
**day 0–2**	0.25 ± 0.06	0.40 ± 0.08
**day 2–4**	0.21 ± 0.02	0.49 ± 0.10
**day 4–7**	0.53 ± 0.04	0.40 ± 0.04
**day 7–9**	0.37 ± 0.06	0.26 ± 0.06
**day 9–11**	0.17 ± 0.03	0.01 ± 0.06
**day 11–14**	-0.10 ± 0.05	-0.02 ± 0.03
**day 14–16**	-0.07 ± 0.09	-0.13 ± 0.09
**day 16–18**	-0.06 ± 0.04	-0.04 ± 0.02
**day 18–22**	-0.12 ± 0.03	-0.16 ± 0.03

Table S3. Content of carbon (C), nitrogen (N) and phosphorus (P) in the algal biomass calculated at day 11 of the first growth experiment (Quantitation of prymnesins in the biomass of two *P. parvum* strains during pH limited growth) where the algae cultures achieved the maximum biomass, (n = 3). 12.4 mg/L N (nitrate, NO_3^-) and 1.12 mg/L P (phosphate, PO_4^{3-}) were added to the media prior to the incubation start. Thus, plenty of nutrients were left in the media at the point where the cultures achieved the maximum biomass at day 11.

Strain	Cell volume [μm³]	C in biomass [mg/mL] [1]	N in biomass [mg/mL] [2]	P in biomass [mg/mL] [2]
K-0081	261 ± 9.04	18.3 ± 0.41	1.76 ± 0.04	0.13 ± < 0.01
K-0374	135 ± 7.19	9.57 ± 0.93	0.92 ± 0.09	0.07 ± 0.01

Calculated for day 11; n = 3;

[1] calculations based on the equation from [8]: $\log(pgC/cell) = -0.642 + 0.899 \cdot \log(V(\mu m^3))$;

[2] calculated with C:N and C:P ratios from [9]

Table S4. Percentage of biomass-associated prymnesin content based on the comparison of the whole cell culture with either the filtrate or supernatant, determined by the liquid chromatographic (LC) – fluorescence detection (FLD) method (n = 3).

Time point	Sample type	Average [%] [1]	Standard deviation [%]	Relative standard deviation [%]
Day 10	Filtrate	82	1	1
	Supernatant	74	13	18
Day 12	Filtrate	84	7	8
	Supernatant	67	9	13

[1] Calculated as the peak area obtained from the filtrate or supernatant sample through the peak area of the whole cell culture sample multiplied with 100 (= % prymnesins in water) and subtracted from 100. Peak area represents the sum of all prymnesins present in the sample obtained after liquid-liquid extraction and fluorescence derivatization using the AccQ-Fluor reagent kit (Waters, Milford, USA).

Table S5. Percentage of the biomass-associated prymnesin content based on the comparison of the whole cell culture with either the filtrate or supernatant determined by the liquid chromatographic (LC) – high resolution mass spectrometric (HRMS) detection method, (n = 3).

Time point	Sample type	Average [%] [1]	Standard deviation [%]	Relative standard deviation [%]
Day 5	Filtrate	96 [2]	2	2
	Supernatant	N/A [3]	N/A [3]	N/A [3]
Day 10	Filtrate	84	3	3
	Supernatant	75	7	10
Day 12	Filtrate	89	6	7
	Supernatant	65	16	24
Day 17	Filtrate	82	11	13
	Supernatant	54	9	17
Day 21	Filtrate	92 [2]	2	2
	Supernatant	63	7	12

[1] Calculated as the peak area obtained from the filtrate or supernatant sample through the peak area of the whole cell culture sample multiplied with 100 (= % prymnesins in water) and subtracted from 100. Peak area represents the sum of extracted ion chromatograms of single and double charged protonated ion species of previously identified B-type prymnesins ± m/z 0.02.

[2] There was almost no HRMS-signal present in the filtrate sample, therefore results are associated with higher uncertainty.

[3] N/A; not available, no pellet was visible after centrifugation.

References

1. Pierrot, D.; Lewis, E.; Wallace, D.W.R. MS Excel program developed for CO_2 system calculations. ORNL/CDIAC-105a. Carbon Dioxide Information Analysis Center, Oak Ridge National Laboratory, U.S. Department of Energy: Oak Ridge, TN, 2006, doi: 10.3334/CDIAC/otg.CO2SYS_XLS_CDIAC105a
2. Lewis, E.; Wallace, D.W.R. Program Developed for CO_2 System Calculations. ORNL/CDIAC-105. Carbon Dioxide Information Analysis Center, Oak Ridge National Laboratory, U.S. Department of Energy: Oak Ridge, TN, USA 1998.
3. Mehrbach, C.; Culberson, C.H.; Hawley, J.E.; Pytkowicz, R.M. Measurements of the apparent dissociation constants of carbonic acid in seawater at atmospheric pressure. *Limnol. Oceanogr.* **1973**, *18*, 897–907.
4. Dickson, A.G.; Millero, F.J. A comparison of the equilibrium constants for the dissociation of carbonic acid in seawater media. *Deep-Sea Res.* **1987**, 34, 1733–1743.
5. Rasmussen, S.A.; Meier, S.; Andersen, N.G.; Blossom, H.E.; Duus, J.Ø.; Nielsen, K.F.; Hansen, P.J.; Larsen, T.O. Chemodiversity of ladder-frame prymnesin polyethers in *Prymnesium parvum*. *J. Nat. Prod.* **2016**, *79*, 2250–2256, doi:10.1021/acs.jnatprod.6b00345.
6. Hems, E.S.; Wagstaff, B.A.; Saalbach, G.; Field, R.A. CuAAC click chemistry for enhanced detection of novel alkyne-based natural product toxins. *Chem. Commun.* **2018**, *54*, 12234, doi:10.1039/c8cc05113e.
7. Binzer, S.B.; Svenssen, D.K.; Daugbjerg, N.; Alves-de-Souza, C.; Pinto, E.; Hansen, P.J.; Larsen, T.O.; Varga, E. A-, B- and C-type prymnesins are clade specific compounds and chemotaxonomic markers in *Prymnesium parvum*. *Harmful Algae* **2019**, *81*, 10–17, doi:10.1016/J.HAL.2018.11.010.
8. Menden-Deuer, S.; Lessard, E.J. Carbon to volume relationships for dinoflagellates, diatoms, and other protist plankton. *Limnol. Oceanogr.* **2000**, *45*, 569–579.
9. Johansson, N.; Granéli, E. Influence of different nutrient conditions on cell density, chemical composition and toxicity of *Prymnesium parvum* (Haptophyta) in semi-continuous cultures. *J Exp. Marine Biol. Ecol.* **1999**, *239*, 243–258.
